# Rituximab Hypersensitivity: From Clinical Presentation to Management

**DOI:** 10.3389/fphar.2020.572863

**Published:** 2020-09-08

**Authors:** Ghada E. Fouda, Sevim Bavbek

**Affiliations:** ^1^ Allergy and Immunology Center, Al-Azhar University, Cairo, Egypt; ^2^ Division of Immunology and Allergy, Department of Chest Diseases, Ankara University School of Medicine, Ankara, Turkey

**Keywords:** biologic agents, rituximab, hypersensitivity, desensitization, drug allergy, serum sickness, monoclonal antibody, infusion reaction

## Abstract

Rituximab is a chimeric monoclonal antibody (mAb) against CD20 molecule which is expressed on human B cells. It has been used for the treatment of various lymphoid malignancies, lymphoproliferative diseases, and rheumatologic disorders. Rituximab is generally well tolerated. However, increased use of rituximab has been associated with hypersensitivity reactions (HSRs), which can be classified as infusion-related, cytokine-release, type I (IgE/non-IgE), mixed, type III, and type IV reactions. Immediate infusion-related reactions to rituximab are quite common and decrease in frequency with subsequent infusions. However, in about 10% of patients, severe infusion-related reactions develop, which prevent its use. Some of the immediate infusion reactions are due to a cytokine-release but some reactions raise concerns for type I (IgE/non-IgE) hypersensitivity. Recent studies have shown the presence of serum anti-rituximab antibodies, either represented by the IgG or IgE isotype. In some cases, clinical manifestations of IgE-mediated reactions and cytokine-release reactions partially overlap, which is called a mixed reaction. Classified as Type III reaction, rituximab-induced serum sickness reactions have been reported in patients with autoimmune diseases and hematological malignancies. The classic serum sickness triad (fever, rash, and arthralgia) has been observed in patients mainly with an underlying rheumatologic condition. Severe delayed type IV hypersensitivity reactions including non-severe maculopapular rash to severe reactions such as Stevens–Johnson syndrome and toxic epidermal necrolysis have been rarely reported following rituximab injection. Comprehensive reviews focused on rituximab-induced HSRs are scarce. We aimed to review clinical presentations, underlying mechanisms of rituximab hypersensitivity, as well as management including rapid drug desensitization.

## Introduction

Monoclonal antibodies (mAbs) have become mandatory for neoplastic targeted therapies as well as chronic inflammatory and autoimmune diseases (Beck et al., 2010; Li et al., 2013; Patel and Khan, 2017; Picard and Galvão, 2017; Özyiğit et al., 2020). Rituximab is a chimeric IgG mAb directed against CD20 antigen that is expressed on normal and malignant B cells (Plosker and Figgitt, 2003). It was initially approved as an anti-neoplastic agent (Vikse et al., 2019). Later on, it became a bright treatment opportunity instead of the conventional treatment for chronic granulomatosis and inflammatory diseases (Wong and Long, 2017).

Rituximab treatment results in two main categories of adverse reactions, including immunodeficiency and hypersensitivity reactions (HSRs). The drug is considered to be of good and accepted tolerability, (Vidal et al., 2011; Makatsori et al., 2014); however, increased use of rituximab has been associated with HSRs (Brennan et al., 2009; Patel and Khan, 2017; Picard and Galvão, 2017; Isabwe et al., 2018). Comprehensive reviews focused on rituximab-induced HSRs are scarce. This article aimed to review and describe clinical presentations, underlying mechanisms of rituximab hypersensitivity as well as management including rapid drug desensitization (RDD).

## Hypersensitivity Reactions to Rituximab

### Classification and Prevalence

Adverse drug reactions are classified into two major types: type A reactions are common and may occur in any individual. Type B reaction, also called “Drug Hypersensitivity”, is uncommon, unpredictable, and occurs only in susceptible individuals. Drug Hypersensitivity occurs *via* immune (allergic) or non-immune mechanisms. Allergic drug reactions can manifest with many different clinical presentations, and to better explain these variations in clinical presentations, traditional Gell and Coombs classification is used (Pichler, 2006).

Like other pharmaceutical agents, biological agents can cause adverse drug reactions. Because of the inherent differences between biologicals and pharmaceutical drugs, adverse reactions to biologicals cannot be classified according to the traditional classification. Therefore, alternative classification schemes have been suggested. One proposal by Pichler is based on the immunological activity of biologicals and to distinguish it from the classification of adverse drug reactions, the Greek alphabets, alfa, beta, gamma, delta, and epsilon have been used. This classification includes five groups: (Type *α*) high cytokine levels, (Type *β*) hypersensitivity reactions, IgE, IgG, and T cell mediate reactions, (Type *δ*) immune imbalance syndrome, (Type *γ*) cross-reactivity with native proteins, and (Type *ε*) non-immunologic adverse effects (Pichler, 2006; Patel and Khan, 2017; Wong and Long, 2017). Alfa reactions are associated with high cytokine levels, and in most cases, high cytokine levels occur due to endogenous cell activation, that is called a cytokine release reaction. Beta-type reactions are HSRs and are further defined as immediate and delayed and occur with IgE, IgG, and complement or T cell involvement. (Pichler, 2006; Corominas et al., 2014; Picard and Galvão, 2017).

However, recently a new classification was proposed considering phenotypes, endotypes, and biomarkers indicating underlying endotype. Phenotype and endotypes were based on the clinical presentation, the cells and mediators involved in the reaction respectively. The classification proposes four patterns of phenotypes: type I reactions (IgE/non-IgE), cytokine-release reactions, mixed reactions (type I/cytokine-release), and delayed-type IV reactions (Isabwe et al., 2018) (**Table 1**). This new classification encompasses the classic HSRs described by Gell and Coombs as well as reactions outside the classification, but it has no space for type II or III reactions since this classification focuses on the reactions that might benefit from desensitization.

**Table 1:** Classifications of hypersensitivity reactions to biological agents including rituximab.

Type of reaction	Mechanism/Diagnostic criteria	References No≠
Infusion-related reaction	Non-immunologic, monocytes, macrophages, cytotoxic T cells, natural killer cells activation/clinical presentations and course, levels of IL-1, IL-6, and TNF-α	Pichler, 2006; Corominas et al., 2014; Galvão and Castells, 2015; Khan, 2016; Isabwe et al., 2018
Cytokine release reaction	Non-immunologic, monocytes, macrophages, cytotoxic T cells, natural killer cells activation/clinical presentations and course, levels of IL-1, IL-6. and TNF-α	Pichler, 2006; Corominas et al., 2014; Galvão and Castells, 2015; Khan, 2016; Isabwe et al., 2018
Type I reaction (IgE/non-IgE)	IgE or non- IgE dependent mast cell, basophil activation/clinical presentations, level of tryptase, skin tests, BAT	Pichler, 2006; Corominas et al., 2014; Galvão and Castells, 2015; Khan, 2016; Isabwe et al., 2018
Mixed reaction	IgE or non-IgE dependent mast cell, basophil activation and monocytes, macrophages, cytotoxic T cells, natural killer cells activation/clinical presentations, skin tests, BAT, levels of tryptase, IL-1, IL-6, and TNF-α	Pichler, 2006; Corominas et al., 2014; Galvão and Castells, 2015; Khan, 2016; Isabwe et al., 2018
Type III reaction	Not clear, may be related to C-fixing IgM and IgG antibodies and Fc-IgG receptor-mediated neutrophil activation clinical presentations, RF, immunoglobulins, and HACA levels	Karmacharya et al., 2015
Type IV reaction	T-cell mediated or other mechanisms/clinical presentations, immunohistological examination	Henning and Firoz, 2011; Macdonald et al., 2015; Fallon and Heck, 2015; Chen et al., 2018

C, complement; HACA, human anti-chimeric antibody; TNF-α, tumor necrosis factor-α.

#### Infusion-Related Reactions

Patients mostly suffer from common acute infusion reactions that occur in a short time after infusion. Although the pathogenesis of these reactions is not very clear, it’s usually affected by the rate of infusion, pointing out to the possibility of a non-immunologic mechanism and the role of the inflammatory cytokines such as IL-6 and tumor necrosis factor-*α* (Khan, 2016; Patel and Khan, 2017). Clinical presentations resemble type I or cytokine-release, but they are mild to moderate in severity and subside gradually with the following infusions (Plosker and Figgitt, 2003; Galvão and Castells, 2015).

#### Cytokine Release Reactions

The phenotype is defined as fever/chills, nausea, pain, headache, and rigors not responding to premedication/slower infusion rate during the first infusion. Clinical symptoms and signs are usually due to the cytokine release that is characterized by elevated serum TNF-α and IL-6 levels at the time of the reaction compared with their normal baseline (Isabwe et al., 2018).

#### Type I Reactions (IgE/Non-IgE)

The reaction is defined as flushing, pruritus, urticaria, shortness of breath, wheezing, hypotension, and life-threatening anaphylaxis. Reactions are associated with IgE or non-IgE mediated mast cell/basophil degranulation leading to massive histamine, leukotrienes and prostaglandins release. Skin test positivity and/or specific IgE to rituximab is indicative of both IgE-mediated and mixed reactions (Patel and Khan, 2017; Wong and Long, 2017; Isabwe et al., 2018).

#### Mixed Reactions

Mixed reactions are a combination of cytokine release and IgE-mediated reactions. Clinical presentations are characterized by wheezing, flushing, urticaria, pruritus, with fever/chills, nausea, pain, headache, and rigor. Skin test positivity and/or specific IgE to rituximab as well as increased levels of tryptase, IL-1, IL-6 and TNF-α can occur (Patel and Khan, 2017; Isabwe et al., 2018).

There are limited data on the frequency of HSRs and standard infusion reactions to rituximab. Additionally, the lack of consensus on the definition and classification of HSRs makes the data even confusing (**Table 2**). Among biological agents, rituximab has the highest reported infusion reactions, with up to 77% reported with the first infusion (van Vollenhoven et al., 2013). It also has a relatively high rate of HSRs, consistent with the IgE-mediated reactions, reported with 5 to 10% of infusions (Brennan et al., 2009; Galvão and Castells, 2015). In a study by Isabwe et al., prevalence of type I, cytokine-release, mixed type, and delayed-type IV reactions were reported as 63, 13, 21, and 3% respectively (Isabwe et al., 2018).

**Table 2:** Prevalence and severity of HSRs to rituximab.

References	Types, clinical features, and severity	
Brennan et al., 2009 n = 14 patients	**Clinical presentations**	Approximately 57% cutaneous, 50% cardiovascular, 50% respiratory, 30% fever, 30% throat, 20% gastrointestinal, and 30% neurological reactions
	**Severity**	Grade I: 25%, Grade II: 50%, Grade III: 30%
van Vollenhoven et al., 2013 n = 3194 patients	-Acute infusion related reactions: 77% of patients	
Levin et al., 2017 n = 67 patients	**Incidence of reactions in relation to the number of infusions**	63% of reactions during 1^st^ infusion, 9% during 2^nd^ infusion, 15% during cycles 3–10, 7% during cycles 11–20, 6% during cycles 20–53
	**Clinical presentations**	63% cutaneous reactions, 45% generalized pruritis, 21% flushing, 16% hives
	**Severity**	Grade 1A: 18%, Grade 1B: 9%, Grade 2: 61%, Grade 3: 10%, Grade 4: Less than 1%
Isabwe et al., 2018 n = 52 patients	**Reactions**	Acute infusion reactions: 20-50% Type I reactions: 63% Cytokine-release reactions: 13% Mixed reactions: 21% Delayed reactions: 3%
	**Severity**	Grade I: 13%, Grade II: 60%, Grade III: 29%
Görgülü et al., 2019 n = 24 patients	**Reactions** (Based on Isabwe et al., 2018)	Type I (IgE/non-IgE): 46% Cytokine-release reaction: 12.5% Mixed reactions: 41.5%
	**Clinical presentations**	Cutaneous symptoms 92%, Respiratory symptoms 88%, Cardiovascular symptoms 67%, Gastrointestinal symptoms 55%, Neurologic/muscular symptoms 29%, Fever (≥38.3°C) 46%
	**Severity**	Grade I: 0%, Grade II: 63%, Grade III: 37%

Brown classification is commonly used in the classification of severity of HSRs to mAbs. Grade 1 (mild) represents only skin/subcutaneous involvement, Grade 2 (moderate) presents gastrointestinal, cardiovascular or respiratory system affection, and Grade 3 (severe) consisted of failure of neurologic, respiratory, or cardiovascular systems (Brown, 2004).

#### Serum Sickness Reactions

Rituximab-induced serum sickness (RISS, type III) reactions have been observed less commonly. A systematic review reported 33 cases from 25 articles, the majority with underlying rheumatoid diseases. However, the systematic review has limitations such as the lack of confirmatory tests in all cases. Although the pathogenesis is not clear, it seems to be related to complement-fixing IgM and IgG antibodies targeted at an immunogenic part of the drug. The typical presentation has been found in 48.5% of cases. Symptoms are usually benign and self-limited in mild cases. Corticosteroid treatment may be beneficial, but premedication is not always effective. Correct diagnosis of RISS remains an unmet need (Karmacharya et al., 2015).

#### Type IV Reactions

Delayed type IV reactions are mostly presented with a maculopapular rash (Macdonald et al., 2015). Severe cutaneous reactions may occur, but they are probably rare. Two cases of Stevens–Johnson syndrome (SJS), one case of toxic epidermal necrolysis (TEN), and two cases of SJS-TEN caused by rituximab have been reported in a review (Chen et al., 2018). The U.S. FDA adverse events report seven cases of rituximab induced TEN (Fallon and Heck, 2015). There might be a false diagnosis of a case of SJS due to similarities in clinical findings, pathology, and prognosis resembling paraneoplastic pemphigus (PNP). To confirm cases of SJS/TEN in rituximab and differentiate them from PNP, direct and indirect immunofluorescence could be used (Joly et al., 2000; Henning and Firoz, 2011).

## Diagnosis

The detailed clinical history is crucial for determining the type and severity of the HSR (Picard and Galvão, 2017; Yang and Castells, 2019; Görgülü et al., 2019). Different hypersensitivity mechanisms such as type I IgE or non-IgE reactions could give rise to the same clinical picture (Isabwe et al., 2018). *In vivo* tests such as skin prick test (SPT) and intradermal testing (IDT), drug provocation test (DPT), and *in vitro* tests including specific IgE, basophil activation test (BAT), serum levels of tryptase, IL-1, IL-6, TNF-α, or lymphocyte transformation test (LTT) are used to define the phenotype of the HSR. Clinical history, *in vivo* and *in vitro* tests are all essential for personalized and precision medicine, but there is remarkable heterogeneity on diagnostic approaches (Santos and Galvao, 2017; Isabwe et al., 2018; Madrigal-Burgaleta et al., 2020).

### Skin Testing as Diagnostic and Predictor for Breakthrough Reactions During Desensitization

Skin testing is the primary step for assessing HSRs to rituximab (Brennan et al., 2009). Skin test positivity demonstrated through SPT or IDT to rituximab suggests an IgE-mediated reaction. Despite insufficient evidence for optimal timing, skin testing with the culprit drug can be done within 2–4 weeks following the reaction to avoid false negative results (Alvarez-Cuesta et al., 2015; Santos and Galvao, 2017; Isabwe et al., 2018; Madrigal-Burgaleta et al., 2020). SPT is done using a drop of concentrated rituximab 10 mg/ml, and if it is negative, IDT is then performed with dilutions from 1:1,000 up to 1:1 (Wong and Long, 2017; Santos and Galvao, 2017; Isabwe et al., 2018). Positivity to rituximab is usually seen with IDT more than SPT. In a study, 30% of patients with HSRs to rituximab were positive in IDTs, and none of them were positive in SPT (Görgülü et al., 2019). Similarly, in an early study, IDT for rituximab was positive in six out of nine patients (Brennan et al., 2009). However, in another study, 20% of the patients were SPT positive, and 32% were IDT positive among 52% of skin test positive patients (Isabwe et al., 2018).

Skin test positivity was found to be correlated with the frequency of respiratory symptoms, but not to the severity of the initial reaction to rituximab (Görgülü et al., 2019). However, Isabwe et al. have found that positive rituximab skin testing was strongly associated with severe initial rituximab HSRs. The percentage of type I breakthrough reactions during desensitization was as high as 69% in patients with a positive skin test. Therefore, skin testing could be helpful in the prediction of the type of breakthrough reactions. On the other hand, breakthrough reactions for skin test negative patients were lower in severity during desensitization (Isabwe et al., 2018). Madrigal-Burgaleta et al. showed that patients with a positive SPT tend to encompass an important percentage of breakthrough reactions during desensitization (Madrigal-Burgaleta et al., 2019). Wong and Long have concluded that there is no significant difference in the risk of a breakthrough reaction if the patient is skin test positive or negative (Wong and Long, 2017). The differences may be due to patient selection, different concentrations and volume used for skin tests, the clinical symptoms of the patients, their severity, the time between the reaction and the study, such differences need to be clarified (Wong and Long, 2017).

### Drug Provocation Test

Data on the use of DPT to biological agents are scarce and come from a few specialized centers. The Ramon y Cajal University Hospital (RCUH) group is the pioneer of DPT with the largest reported series with antineoplastics and biologicals. They positioned the use of DPT in patients with negative or equivocal skin test results provided the risk-assessment is favorable. There are no international guidelines for DPT with these drugs but the RCUH protocol is based on direct re-administration of the culprit drug under standard conditions (Alvarez-Cuesta et al., 2015; Madrigal-Burgaleta et al., 2019; Madrigal-Burgaleta et al., 2020; Martí-Garrido et al., 2020). If patients showed positive skin testing or positive DPT, then the HSR would be confirmed; conversely, a negative DPT would rule out an HSR. Thus, not doing DPTs routinely in the diagnosis of an HSR to biologics could bias the safety or the efficiency of RDD. However, this approach needs trained personnel and well-equipped center that limit its wide implementation (Madrigal-Burgaleta et al., 2019).

### *In Vitro* Tests

In a patient with two immediate reactions to rituximab, non-isotype-specific and sIgE to rituximab were positive in the serum samples. More importantly, rituximab stimulated peripheral blood mononuclear cells displayed a response associated with a Th2 cytokine production profile (Vultaggio et al., 2012). The use of sIgE, BAT, serum levels of tryptase, IL-1, IL-6, TNF-α, and LTT as diagnostic tools to biologicals are restricted to selected patients in expert centers (Madrigal-Burgaleta et al., 2020).

## Management

There’s a general agreement about avoiding rituximab that has caused type IV HSR such as SJS, TEN, EM and DRESS as well as RISS (Hong and Sloane, 2019; Yang and Castells, 2019). For mild to moderate common infusion reactions to rituximab, the manufacturer’s instructions are to reduce the rate of infusion and premedicate the patients with an antihistamine, acetaminophen and methylprednisolone prior to dosing and liaise the allergist if required (https://www.ema.europa.eu/en/documents/product-information/mabthera-epar-product- information_en.pdf). In the event of an HSR to rituximab such as Type 1, cytokine release, mixed reaction and delayed maculopapular rash, RDD is a safe and valid alternative. The management by multidisciplinary teams led by expert allergists and access to adequate facilities for allergy procedures has shown to be the optimal approach, with the best efficacy and safety results (Isabwe et al., 2018; Görgülü et al., 2019; Hong and Sloane, 2019).

### Patient Selection for Rapid Drug Desensitization

Patients with a clinical history of HSR to biologicals and who have a confirmation from their referring specialist to use the drug as their first line of choice with no better alternatives are possible candidates for RDD (Sloane et al., 2016; Patel and Khan, 2017; Picard and Galvão, 2017). Different groups use different criteria for patient selection for RDD. The Brigham and Women’s Hospital (BWH), a very well-known center for RDD, recommends that RDD should always be performed on patients with positive *in vivo*/*in vitro* tests, regardless of the grade of the initial HSRs. If the test restults are negative and the initial HSR is Grade I (low risk), a challenge may be performed. If there is no reaction during the challenge, the patient can be sent back to regular infusion. However, if there is a reaction, RDD should be performed for the next drug exposure. If the test results are negative and the initial HSR is Grade II/III (moderate-high risk), RDD is indicated (Castells, 2009; Galvão and Castells, 2015; Isabwe et al., 2018). RCUH considers that DPT should always be performed systematically prior to RDD, but only if the risk-assessment for the patient is favorable. This risk-assessment strategy involves a number of factors (*e.g.* ST, serum biomarkers, patient comorbidities, and patient wishes) and is discussed in a collaborative decision-making process including the referring physician, the allergist, and the patient (who makes the final decision) (Alvarez-Cuesta et al., 2015; Madrigal-Burgaleta et al., 2019).

### Premedication in Rapid Drug Desensitization

Premedication is a controversial issue and there is no categorical recommendation. BWH recommends routine pre-medications in all desensitization protocols such as cetirizine 10 mg, montelukast 10 mg, or zileuton to prevent bronchospasm, famotidine 20 mg for H1 and H2 blockage, and aspirin 81 to 325 mg to prevent flushing caused by prostaglandins. As well as ibuprofen 200 to 800 mg, meperidine 25 mg, or acetaminophen 650 mg for prevention of rigors, pain, and fever. 40 to 50 mg methylprednisolone or other steroids are needed in cases of more severe reactions. Benzodiazepines can be added to control anxiety (Brennan et al., 2009; Castells, 2009; Görgülü et al., 2019; Yang and Castells, 2019).

Contrary, RCUH Allergy Division Desensitization Program showed that in a limited number of patients with confirmed hypersensitivity to Paclitaxel, desensitization alone might be more than enough to control allergic reactions, and premedication with antihistamines and corticosteroids made no difference to the breakthrough reactions (Lopez-Gonzalez et al., 2018). The same group currently reported that they used only standard premedication for each drug (according to prescribing information by the manufacturer and institutional protocols) but for some cases additional premedication customized to their initial or breakthrough reactions (Madrigal-Burgaleta et al., 2019). Their results show no significant increase in the number or severity of breakthrough reactions when compared with results reported by other groups (Brennan et al., 2009; Sloane et al., 2016; Picard and Galvão, 2017; Isabwe et al., 2018).

Even if evidence is weak and the topic is controversial, whenever possible, *ß*-blockers and ACE-Inhibitors should be avoided one day prior to desensitization as the former blocks the action of epinephrine and the latter may even aggravate immediate reaction (Aberer et al., 2003; Lebel et al., 2016; Görgülü et al., 2019; Madrigal-Burgaleta et al., 2019; Yang and Castells, 2019).

Available data suggest that systematic use of premedication may not play a significant role in improving the effectiveness and safety of RDD and should be carefully and individually discussed if their only purpose is to prevent breakthrough reactions. All groups seem to be recommended a personalized approach and further studies are needed for optimal premedication protocols (**Table 4**).

### Rapid Desensitization Protocols

Several protocols have been proposed (Puchner et al., 2001; Jerath et al., 2009; Amorós-Reboredo et al., 2015; Madrigal-Burgaleta et al., 2019). However, the protocol developed by the BWH has gained wide acceptance. The 12-step protocol consists of three bags, where tolerance to the offending antigen dose is obtained by giving the patient 2 to 2.5 incremental doses of the drug, through increasing the rate of infusion and the concentration of the drug at fixed 15-min intervals. The remaining amount of the total dose is infused at a steady rate of infusion in the last step. A 4-bag, 16-step protocol can be initiated for more severe reactions (Castells, 2009). Few studies specifically focused on rituximab desensitization (**Table 3**) (Brennan et al., 2009; Amorós-Reboredo et al., 2015; Ataca et al., 2015; Tal et al., 2016; Wong and Long, 2017; Görgülü et al., 2019). A recent study with 141 RDD in reported that only 14 RDD were interrupted by breakthrough reactions leading to the incompletion of two desensitizations only due to development of anaphylaxis, with a success rate for RDD of 98.5%. Usually, breakthrough reactions are mild and develop in the last steps of the protocol (Görgülü et al., 2019). In another study, five patients have had 19 desensitizations to rituximab where all RDDs were successful. Only two patients have developed breakthrough reactions in the form of neuromuscular reactions. Two out of five patients had positive IDT (Amorós-Reboredo et al., 2015). Wong and Long have demonstrated that out of 25 patients, 29% have experienced breakthrough reactions. IDTs only were positive in five of 18 patients. All 170 RDDs were conducted successfully using high-risk, intermediate or rapid protocols that consisted of 3–8 steps (Wong and Long, 2017). Brennan and colleagues have successfuly completed all 55 RDDs to rituximab for 14 patients with a 12-step protocol, where 40% of patients had symptoms of grade 1 breakthrough reactions. Only nine patients underwent IDT showing six positive results (Brennan et al., 2009). A different study has performed 53 RDDs on seven patients without skin testing. They used a modified 12-step protocol with 100% success. Grade 1 breakthrough reactions were reported in three RDDs (Tal et al., 2016).

**Table 3:** Rapid desensitization protocols data from different studies.

Reference	Patients/Desensitization number	RDD protocol Success rate	BTRs	Skin test positivity
Brennan et al., 2009	14/55	12-step protocol 100% successful	40% of patients Grade 1 reaction	IDT: 6/9 patients
Amorós-Reboredo et al., 2015	5/19	12-step protocol 100% successful	2 patients had neuromuscular reactions	IDT: 2/5 patients
Tal et al., 2016	7/53	Modified 12-step protocol 100% successful	Grade 1 reaction in 3 RDDs	ND
Wong and Long, 2017	25/170	3 protocols: High-risk, intermediate and rapid protocols (3-8 steps) 100% successful	29% of patients (53% Grade 1 37% Grade 2 9% Grade 3)	IDT: 5/18 patients
Görgülü et al., 2019	24/141	12-step protocol 16-step in patients with severe reactions 98.5% successful	14 patients -Grade 1: 17% -Grade 2: 33% -Grade 3: 8% -2 RDDs couldn’t be completed	IDT: 6/20patients
Madrigal-Burgaleta et al., 2019*	21/130	10-step protocol 100% successful*	Not reported	Not reported

BTRs, Break Through Reactions; NP, No data; IDT, Intradermal test.

*Data was not provided specifically on the rituximab-reactive patients but extracted from biological patients as a whole.

### Management of Breakthrough Reactions During Desensitization

The rapid desensitization protocol does not need to be suspended because of a breakthrough reaction. Once the reaction is controlled, the RDD protocol can be reinitiated and followed to completion.

Breakthrough reactions to rituximab were generally mild. However, moderate and severe reactions may appear, although less frequently than mild reactions, and the majority of desensitizations were completed (Lebel et al., 2016; Görgülü et al., 2019). Cutaneous involvement has been the main feature of breakthrough reactions (Brennan et al., 2009; Görgülü et al., 2019). If a breakthrough reaction occurs, the infusion must be immediately stopped, and specific medications should be given according to the symptoms experienced. For future RDD, protocols should be customized based on the severity, symptoms and type of breakthrough reaction such as additional premedication and/or dilutions (Madrigal-Burgaleta et al., 2019; Yang and Castells, 2019).

## Conclusion

Although rituximab is generally well tolerated, its widespread use has entailed an increase in the number of HSRs. There are different proposed classifications for HSRs to mAbs including rituximab with some degree of overlap. Each type of HSR has its features, course, and management. The new proposed classification seems to have clinical implication in terms of a personalized and precise approach. Skin tests, done 2–4 weeks after reaction, is the first step in the diagnostic algorithm and in case of negativity, should be followed by DPT in the availability of adequate settings. RDD is considered a cornerstone of treatment for patients with immediate-type HSRs to rituximab, whereas it seems to be performed in few centers. Therefore, desensitization approach needs more awareness and needs to gain more acceptance. Overall, institutional multidisciplinary teams promoted by allergists to manage HSRs to mAbs including rituximab is crucial.

## Perspectives

New mAbs targeting CD20, obinutuzumab, ofatumumab, veltuzumab and ocrelizumab have been currently introduced to the market. Infusion reactions are the most common adverse event reported with these anti-CD20 mAbs, whether chimeric, humanized or human. However, the relative frequencies have not been studied in a head-to-head fashion. There is no data about cross-reactivity between these mAbs. This may be related to the exclusion of patients with a history of severe allergic reactions to these mAbs from such studies (Gelfand et al., 2017; Salles et al., 2017) (**Table 4**).

**Table 4:** Unmet needs and future research for HSRs to rituximab.

**Classification**	Clear and acceptable classification for the type of reaction
	Well defined criteria for this classification
	Clear clinical and laboratory criteria to differentiate the type of reactions
	Effective biomarkers for clear endotyping underlying reactions
**Epidemiology**	Lack of data about morbidity and mortality of each type of reactions
RISS*	Clinical trials in such patients with allergist involvement to obtain better evidence for the diagnostic criteria and management
SCARs**	Clearly understanding the mechanism and diagnostic methods
	The differential diagnosis in case of exposure to other concomitant medications that are known to cause SCARs
**Skin tests**	Non-irritant dose of rituximab
	The high cost of rituximab to use for skin tests
	Lack of skin test reagents containing all the immune epitopes
	Perfect timing for doing skin tests after an HSR
	Role of skin test in the prediction of breakthrough reactions
	The role of patch test in the diagnosis of delayed-type reaction
***In vitro* tests**	Their roles in diagnosis
**Desensitization**	Optimal premedication protocols
	Candidates for desensitization
**Cross-reactivity**	Between rituximab, obinutuzumab, ofatumumab, veltuzumab and ocrelizumab
**OVERALL**	A multidisciplinary team study including allergists, pharmacologists, nurses, oncologists, hematologists, and other specialties to improve the diagnostic approach and management of HSR s to mAbs and to overcome the unmet needs

*Rituximab induced serum sickness, **Severe cutaneous adverse reaction.

## Unmet Needs and Future Research

There are several unmet needs (**Table 4**) that will lead to future research in this area.

## Author Contributions

SB directed the writing and was responsible for the overall guidance. GF and SB wrote and revised the manuscript.

## Conflict of Interest

The authors declare that the research was conducted in the absence of any commercial or financial relationships that could be construed as a potential conflict of interest.
